# Monte Carlo vs. Pencil Beam based optimization of stereotactic lung IMRT

**DOI:** 10.1186/1748-717X-4-64

**Published:** 2009-12-12

**Authors:** Marcin Sikora, Jan Muzik, Matthias Söhn, Martin Weinmann, Markus Alber

**Affiliations:** 1 Section for Biomedical Physics, University Hospital for Radiation Oncology, Hoppe-Seyler-Str. 3, 72076 Tübingen, Germany

## Abstract

**Background:**

The purpose of the present study is to compare finite size pencil beam (fsPB) and Monte Carlo (MC) based optimization of lung intensity-modulated stereotactic radiotherapy (lung IMSRT).

**Materials and methods:**

A fsPB and a MC algorithm as implemented in a biological IMRT planning system were validated by film measurements in a static lung phantom. Then, they were applied for static lung IMSRT planning based on three different geometrical patient models (one phase static CT, density overwrite one phase static CT, average CT) of the same patient. Both 6 and 15 MV beam energies were used. The resulting treatment plans were compared by how well they fulfilled the prescribed optimization constraints both for the dose distributions calculated on the static patient models and for the accumulated dose, recalculated with MC on each of 8 CTs of a 4DCT set.

**Results:**

In the phantom measurements, the MC dose engine showed discrepancies < 2%, while the fsPB dose engine showed discrepancies of up to 8% in the presence of lateral electron disequilibrium in the target. In the patient plan optimization, this translates into violations of organ at risk constraints and unpredictable target doses for the fsPB optimized plans. For the 4D MC recalculated dose distribution, MC optimized plans always underestimate the target doses, but the organ at risk doses were comparable. The results depend on the static patient model, and the smallest discrepancy was found for the MC optimized plan on the density overwrite one phase static CT model.

**Conclusions:**

It is feasible to employ the MC dose engine for optimization of lung IMSRT and the plans are superior to fsPB. Use of static patient models introduces a bias in the MC dose distribution compared to the 4D MC recalculated dose, but this bias is predictable and therefore MC based optimization on static patient models is considered safe.

## Background

Optimization of stereotactic lung intensity-modulated radiotherapy (lung IMSRT) is challenging for two reasons. First, because of uncertainties in dose calculation in the presence of tissue interfaces (between lung and tumour), and the commonly small fields. Second, because of the uncertainties in dose calculation when optimizing on a static patient model representing a moving target.

Although it has been well documented that conventional (Pencil beam (PB), superposition/convolution) algorithms fail to some degree when calculating dose to lung [1-12], most treatment planning systems (TPSs) use these algorithms both for optimization of lung IMSRT and for final dose calculation, and MC only as a benchmarking tool. Fraass et al [13] have stated that the use of MC dose calculation algorithms for clinical planning improves the dose accuracy in heterogeneous regions of lung and bony anatomy, in particular when applying very small field sizes which exhibit lateral electron disequilibrium effects. Ideally, a TPS comprises a MC dose engine, since an inaccurate dose algorithm will not only introduce dose errors, but will also lead to wrongly optimized treatment plans.

The main argument for conventional algorithms has been that the MC dose calculation algorithms are too slow, especially for use in optimization of IMSRT [14]. However, with the computational power offered by modern computers combined with an efficient MC system [15,16], it should be feasible to use MC not only for recalculation of IMSRT treatment plans but also for optimization itself. The biological IMRT MC-TPS HYPERION[17,18] has been used clinically since 2002. The current version allows for optimization based on both fsPB and MC. In case of MC, it uses a Virtual Source Model (VSM) of the accelerator head [15,16] together with the XVMC dose engine for simulation in the patient [19].

In this study, we first compare our conventional fsPB and MC algorithms to measurements in a lung phantom in order to benchmark their overall accuracy. Then, we apply both algorithms in optimization of lung IMSRT to investigate if direct optimization with MC can provide an advantage over optimization with a conventional algorithm and recalculation with MC.

The comparison of MC with conventional algorithms is not independent of the patient model. Commonly, lung IMSRT is performed on a static model of the moving target and different approaches are used to represent the distribution of densities within the Planning Target Volume (PTV). In this study, we therefore also investigate the influence of various patient models of the lung patient geometry on the results of optimization and compare these results to the real 4D accumulated dose, recalculated with MC on each of 8 computer tomography scans (CTs) representing 8 breathing phases [20].

## Materials and methods

### Verification of the MC and fsPB dose algorithms in a lung phantom

The MC and fsPB algorithms investigated in this study have been presented in detail elsewhere (see [19,16] and [21,22], respectively). Both algorithms calculate dose-to-tissue, since the fsPB is commissioned based on the result of simulation with the MC algorithm.

#### The lung phantom

**Figure 1:**
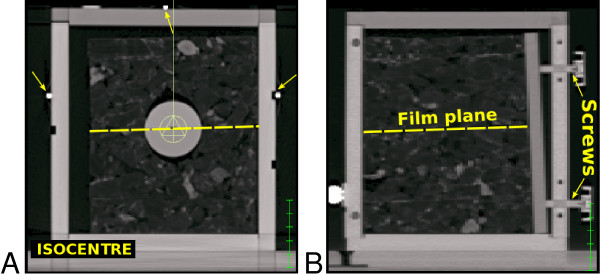
**Lung phantom**. Cross sections through the CT scan of the lung phantom which consisted of a low density cork cube surrounding a homogeneous plastic sphere of variable diameter (here: 4.2 *cm*). The cork cube densities ranged from *ρ*_*min *_= 0.001 to *ρ*_*max *_= 1.09  with an average density of *ρ*_*mean *_= 0.12 , while the plastic sphere had a density of *ρ *= 1.1 . A film could be positioned through the phantom as marked with a dashed line in A and B. The cork cube, plastic sphere and film were fixed relatively to the high density positioning markers (indicated by arrows on A) on the surface of the plastic container by a plastic plate and four plastic screws as shown in B.

We have used a phantom which models a small tumour surrounded by lung tissue (Figure 1) to represent a typical stereotactic lung case. Three plastic spheres representing tumours of 2.7, 4.2 and 5.0 cm diameter were used. These were inserted (one at the time) into the geometrical center of a cork cube which mimicked the heterogeneous density and composition of lung tissue. A slit in the cork and through the tumour allowed for a film to be inserted into the central plane of the phantom as shown in Figure 1. A CT scan with a slice thickness of 2 mm was acquired of the lung phantom (without film) for each tumour size and used for dose calculation.

#### The lung phantom dose distribution calculation, measurements and 2D dose comparisons

For each tumour size, a conformal plan consisting of one vertical beam was created using 6 MV and 15 MV nominal beam energies, and the dose delivered to the phantom was calculated both with MC and fsPB. The maximum dose in the tumour was below 1 Gy to avoid saturation of the radiographic films.

A fine dose grid of 1 *mm*^3 ^was used and the statistical uncertainty of the MC simulations was set to 0.5%. The presence of the dosimetric film was taken into account in the dose calculations by introducing a 1 mm thick layer filled with a density of *ρ *= 1.1  at the position of the film in the CT scan of the lung phantom (as the CT acquisition was made without the film).

Radiographic Kodak X-Omat V films were used for the measurements. These were cut to fit the dimensions of the phantom and placed into the central plane of the cork cube between the hemispheres of the tumour perpendicular to the beam direction (Figure 1). The cork cube was then tightly fixed to the container and the phantom was positioned with the help of the positioning markers. An uncertainty in the position of the film plane of up to 2 degrees relatively to the beam direction was impossible to avoid because of how the slit in the cork cube was cut (Figure 1). An Elekta Synergy S accelerator was used to irradiate the phantoms. All films were developed at the same time and scanned with a Radlink LaserPro 16 scanner. The optical densities of the films were converted to absolute dose by using a calibration curve created for solid water.

The MC and fsPB dose distributions were compared to the 2D film measurements by using the *γ*-index method [23], with an acceptance criteria of 3% dose difference and 3 mm distance-to-agreement (3%/3 mm). Also the tighter 2%/2 mm criteria was investigated. The artefacts from the positioning markers of the phantom were excluded from the analysis and no attempt was made to correct for the slight uncertainty in the positioning of the film. The in-plane and cross-plane profiles through the isocentre where extracted from the 2D film measurements and compared to the MC and fsPB dose distributions. Additionally, depth dose curves were compared between MC and fsPB. The depth dose curves were not measured due to unprecise alignment and a relatively high density contribution of the film when its plane is located along the beam direction.

### The treatment planning optimization tool

An in-house developed optimization tool, HYPERION[18], was used for treatment planning of lung IMSRT in this study. The optimization process in HYPERION works by *constrained optimization *whereby the tumour cell survival is minimized while side effects to the relevant organs at risk are constrained to the maximum tolerable. Details about the optimization process and the HYPERION cost functions can be found in [24,25]. The cost functions used for the purpose of this study were:

1. a *poisson cell-kill EUD *(equivalent uniform dose) model to maximize tumour control [26],

2. a *parallel complication model *to limit the damage to the lungs by constraining the relative fraction of the organ which can be damaged [24,25],

3. and a *maximum overdosage *constraint to avoid target hotspots and to control maximum organ at risk doses in terms of the root mean square (rms) overdosage above a given threshold dose.

Plan optimization is a two-stage process. In stage I, the fsPB computes dose distributions for a large number of beamlets that constitute the fluence profiles. Then, the weights of these beamlets are optimized to yield the idealized fluence profiles. Stage II starts with an initial segmentation of the fluence profiles into a sequence of deliverable MLC segments. Here, the segment doses can either be obtained from a concatenation of their beamlet doses or by a MC calculation. The weights and shapes of these sequences are optimized as described in [27]. In case a segment changes shape, the MC calculation is re-run. An approach via MC-calculated beamlets meets two obstacles: firstly, for a given incident history density, the point-wise dose uncertainty of a beamlet is greater because the dose is smeared out over a greater volume, which in turn causes instabilities in the optimization. Secondly, by definition a beamlet dose cannot include MLC effects which requires a re-computation of the full segment doses in stage II anyway and leads to wrong (usually too steep) field penumbra in stage I.

### Optimization of lung IMSRT plans with MC and PB

#### Patient data and models

One example patient which had previously been treated with lung IMSRT at the University Hospital of Tübingen was used in the study. The patient had a tumour with a diameter of 2.4 cm which was located posteriorly in the lower right lung. The breathing excursion was 2.9 cm mainly in the cranio-cadual direction. A respiratory-correlated CT (RCCT) dataset was acquired with a Siemens Sensation Open scanner reconstructed with 1 × 1 × 3 mm^3 ^voxel size. The CT dataset was grouped into eight CT sets (0/25/50/75% inhale and 100/75/50/25% exhale), where the 0% inhale CT was used as the planning geometry (the static exhale (planning) CT). Contours of the clinical target volumes (CTVs) from all breathing phases as well as of the organs at risk (OARs) were defined and approved by a radiologist. The planning target volume (PTV) was defined as the internal target volume (ITV), i.e. the union of the CTVs from all breathing phases, expanded by 2 mm in order to account for setup uncertainties. This resulted in a PTV of 47.1 cm^3^, around three times larger than the clinical target volume (CTV).

Treatment planning was based on three different patient models accounting for spatial and temporal variations in density within the PTV:

1. *one phase static CT *: this model uses the PTV and the exhale planning CT without any attempt to correct for density variations,

2. *minimum density overwrite, one phase static CT *: in this model, a density less then 0.4  (an empirical value to avoid erratic fluence modulation caused by density-related underdosage in the target volume) was raised to this value within the PTV on the planning CT to mimic the effective density during free breathing irradiation,

3. *average CT*: this model uses the PTV and a superposition of all RCCTs, such that each voxel has a density equal to the weighted average of the Hounsfield values from all breathing phases.

#### Static lung IMSRT planning

For all three patient models IMSRT plans for both 6 and 15 MV beam energies were optimized with both the fsPB and the MC algorithm. The same constraints and a beam arrangement consisting of eleven beams (with gantry angles of 20, 155, 175, 195, 215, 235, 270, 295, 310, 325 and 345), was used in each case. The PTV was prescribed to receive 55 Gy (EUD) in n = 10 fractions and a maximum overdosage constraint of 2 Gy rms above the prescribed dose was applied to avoid hotspots within the PTV (see table 1). Sparing of the contralateral and ipsilateral lung was obtained by constraining the mean dose to 2 and 9 Gy, respectively, and by constraining the mean damage to a relative volume of maximum 8% and 21%, respectively, estimated with the parallel complication model defined by d_o _= 20 Gy and k = 3 [24,25]. In addition, maximum overdosage constraints of 9 Gy and 21 Gy were applied to the spinal cord and unspecified normal tissue within the skin contour.

**Table 1:** Prescribed and resulting isoeffects/EUDs for all patient models plans optimized with fsPB and MC and recalculated with 4DMC; the recalculated MC dose on the static patient models of the fsPB optimized plans are shown in brackets.

		6 MV				15 MV			
*Quantity type*	*Prescr.*	PB	*4D*	MC	*4D*	PB	*4D*	MC	*4D*
*model 1, one phase static CT*									
target* poisson EUD	*55 Gy*	54.25 (57.94)	*58.86*	53.3	*57.35*	55.32 (52.77)	*55.47*	52.09	*58.82*
target* rms overdosage	*2 Gy*	1.99 (4.17)	*4.48*	1.53	*3.39*	2.02 (0.66)	*1.95*	1.97	*5.83*
lung R, mean dose	*9 Gy*	8.39 (10.58)	*10.81*	9.1	*9.32*	8.46 (10.31)	*10.47*	9	*9.26*
lung R, mean damage	*21%*	20.49 (24.48)	*24.93*	21.68	*22.05*	20.71 (23.9)	*24.21*	21.17	*21.6*
skin, rms overdosage	*0.24 Gy*	0.23 (0.61)	*0.59*	0.24	*0.24*	0.24 (0.52)	*0.52*	0.24	*0.24*

*model 2, minimum density overwrite one phase static CT*									
target* poisson EUD	*55 Gy*	56.47 (57.23)	*57.53*	54.98	*56.2*	55.95 (53.68)	*54.37*	52.47	*56.02*
target* rms overdosage	*2 Gy*	2.05 (3.23)	*2.83*	2.02	*1.8*	2.02 (0.96)	*0.6*	2.07	*2.6*
lung R, mean dose	*9 Gy*	8.13 (9.63)	*9.84*	8.91	*9.13*	8.34 (9.96)	*10.06*	8.71	*8.88*
lung R, mean damage	*21%*	19.9 (22.57)	*23.03*	21.25	*21.66*	20.43 (23.14)	*23.38*	20.59	*20.88*
skin, rms overdosage	*0.24 Gy*	0.23 (0.48)	*0.48*	0.24	*0.23*	0.24 (0.48)	*0.48*	0.24	*0.23*

*model 3, average CT*									
target* poisson EUD	*55 Gy*	55.03 (57.53)	*57.46*	52.63	*55.88*	55.32 (52.57)	*55.01*	51.85	*58.49*
target* rms overdosage	*2 Gy*	1.99 (3.71)	*3.09*	1.12	*1.66*	2 (0.52)	*1.25*	2.01	*4.63*
lung R, mean dose	*9 Gy*	8.43 (10.41)	*10.64*	9.12	*9.33*	8.49 (10.3)	*10.49*	9.04	*9.3*
lung R, mean damage	*21%*	20.56 (24.14)	*24.61*	21.74	*22.13*	20.72 (23.85)	*24.23*	21.21	*21.66*
skin, rms overdosage	*0.24 Gy*	0.24 (0.61)	*0.6*	0.24	*0.23*	0.24 (0.53)	*0.53*	0.24	*0.23*

target* - the target dose is calculated in the PTV for static planning and the CTV for 4DMC recalculation, respectively.

A beamlet size of 4 × 2 mm^2 ^was used and segments smaller than 0.64 cm^2 ^were not allowed. We used a 2.5 × 2.5 × 2.5 mm^3 ^dose calculation grid size and a 3% statistical uncertainty per MLC segment for the MC calculation. After optimization, the fsPB plans were recalculated in the static geometries with the MC dose engine. Additionally, all plans were recalculated by 4DMC.

For 4D MC plan calculation (4DMC), the dose was computed in each of the eight static geometries of the RCCT dataset. These MC doses per instance were weighted according to their share of the breathing cycle and accumulated in a common reference geometry (here, the exhale planning CT) by dose warping derived from deformable registration. Details of the 4DMC recalculation method are described in details in [20]. It is important to notice that the PTV encloses a volume where the tumor can be found with certain probability which depends on the breathing pattern. Therefore, it is not possible to evaluate the real dose to the CTV without employing the 4DMC recalculation where the accumulated dose in the CTV is calculated. In the following, we use a common term - *target volume*, which denotes the PTV for static planning and the CTV for 4DMC recalculation, respectively.

## Results

### Experimental verification of MC and fsPB dose engines

**Figure 2:**
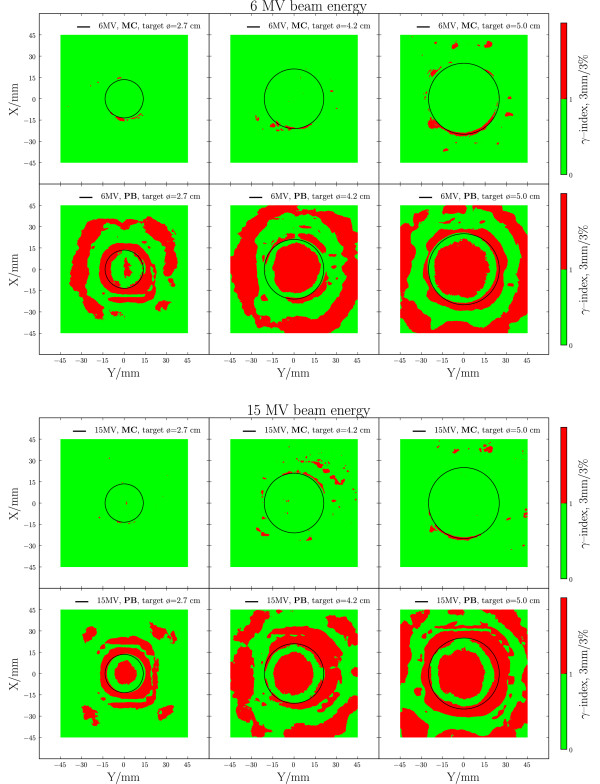
**Verification of dose engines - *γ*-plots**. *γ*-plots of the dose distributions measured with film and calculated with MC and fsPB for all tumour sizes for 6 MV (upper set) and 15 MV (lower set). The acceptance criteria for the *γ *comparisons was set to 3%/3 mm. The tumour outlines are marked by black circles.

**Figure 3:**
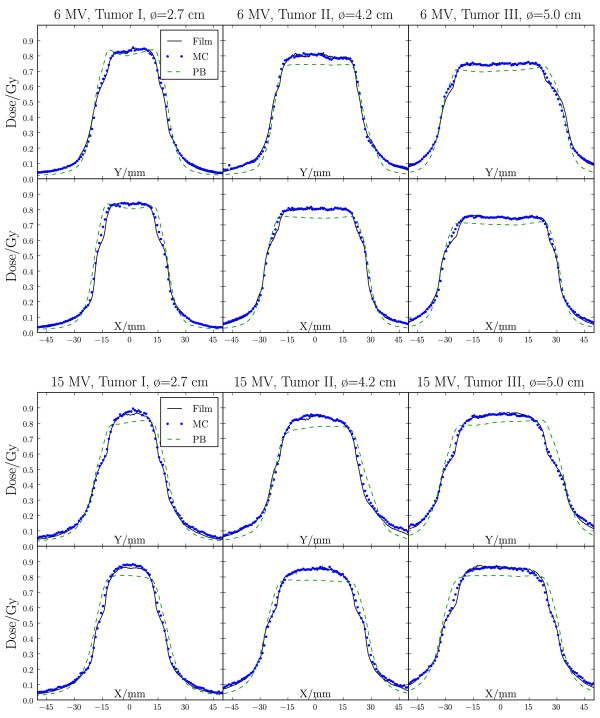
**Verification of dose engines - dose profiles**. 6 MV (upper plots) and 15 MV (lower plots) in-plane (Y) and cross-plane (X) dose profiles as measured with film (solid) and calculated with both MC (dotted) and fsPB (dashed) algorithms for tumour I-III.

**Figure 4:**
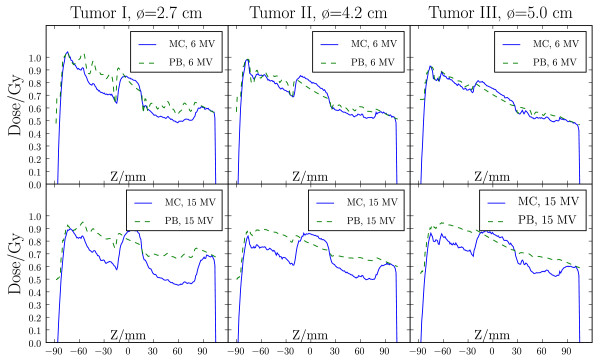
**Verification of dose engines - depth dose curves**. 6 MV (upper row) and 15 MV (lower row) depth dose profiles (Z) calculated with MC (solid) and fsPB (dashed) algorithms for tumour I-III.

The 2D *γ *comparison between the measured and calculated dose distributions is shown in Figure 2 and Table 2 for 6 MV and 15 MV beam energies. Overall, a good agreement was found between MC dose calculation and film measurements, with more than 97% of the points fulfilling the 3%/3 mm acceptance criteria and 84-97% of the points fulfilling the 2%/2 mm criteria. For the fsPB algorithm, the agreement was worse both inside and outside the tumour. Of all points, 45-81% fulfilled the 3%/3 mm acceptance criteria and 25-52% of the points fulfilled the 2%/2 mm criteria. The in-plane and cross-plane profiles (Figure 3) show that the fsPB algorithm underestimates the dose in the target by up to 8%, and produces a too steep penumbra compared to MC and film. The depth dose profile comparison (Figure 4) further shows that MC and fsPB disagree by up to 20% in lung tissue for 15 MV and by up to 15% for 6 MV.

**Table 2:** Agreement between the 2D dose distributions as measured with film and calculated with MC and fsPB for the 3%/3 mm and 2%/2 mm *γ *acceptance criteria; the acceptance (in %) is listed for the whole region (all), the tumour region (tumour) and the region outside the tumour (lung) and for all tumour sizes.

	MC 3%/3 mm			fsPB 3%/3 mm			MC 2%/2 mm			fsPB 2%/2 mm		
Tumour diameter [cm]	all	tumour	lung	all	tumour	lung	all	tumour	lung	all	taret	lung
	6 MV											
2.7	99.6	99.6	99.6	71.8	67.4	72.2	97.3	97.1	97.3	43.3	43.1	43.3
4.2	99.3	99.0	99.4	49.4	31.2	53.1	96.0	92.0	96.8	28.5	23.7	29.5
5.0	97.0	97.2	97.0	46.6	31.3	51.4	89.4	94.4	87.8	30.5	21.2	33.4

	15 MV											
2.7	99.9	99.6	99.9	81.4	60.6	83.0	97.4	96.8	97.4	52.2	41.4	53.0
4.2	97.8	99.6	97.4	51.2	42.4	53.0	84.0	96.7	81.4	25.3	27.7	24.8
5.0	98.3	98.9	98.1	45.4	37.7	47.8	89.8	93.9	88.5	26.6	22.8	27.8

### Static IMSRT dose distributions

**Figure 5:**
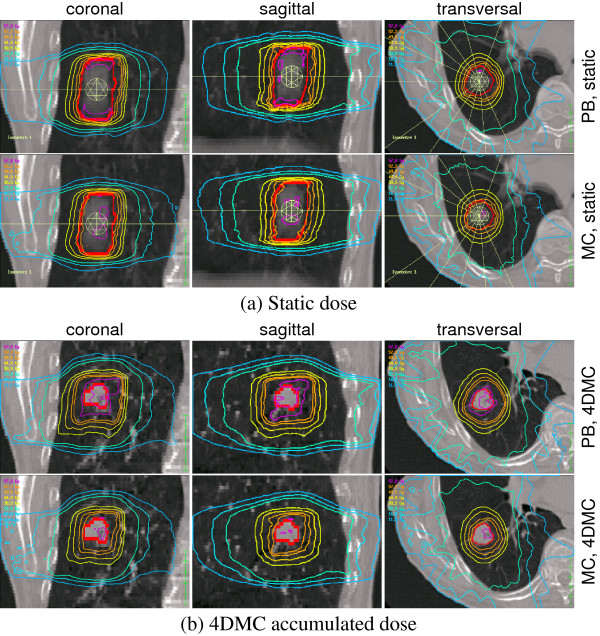
**Dose distribution isodoses**. Example of the resulting dose distributions from optimization with fsPB and MC with 6 MV beam energy and using the average CT patient model (a). The same plans recalculated with 4DMC (b).

An example of the resulting dose distribution from optimization with fsPB and MC is shown in Figure 5, and the prescribed and resulting isoeffects/EUDs for all plans are listed in Table 1. All plans were very close to formally fulfilling the prescribed OAR constraints. The plans optimized with fsPB were also close to fulfilling the prescribed target-EUD for all patient models, while the MC optimized plans resulted in a lower target-EUD than prescribed in all patient models.

The MC recalculation on the static patient models of the fsPB optimized plans shows that most of the prescribed OAR constraints were actually violated. In addition, we found that the fsPB dose calculation algorithm underestimated the target-EUD in case of 6 MV and overestimated the target-EUD in case of 15 MV for all patient models.

**Figure 6:**
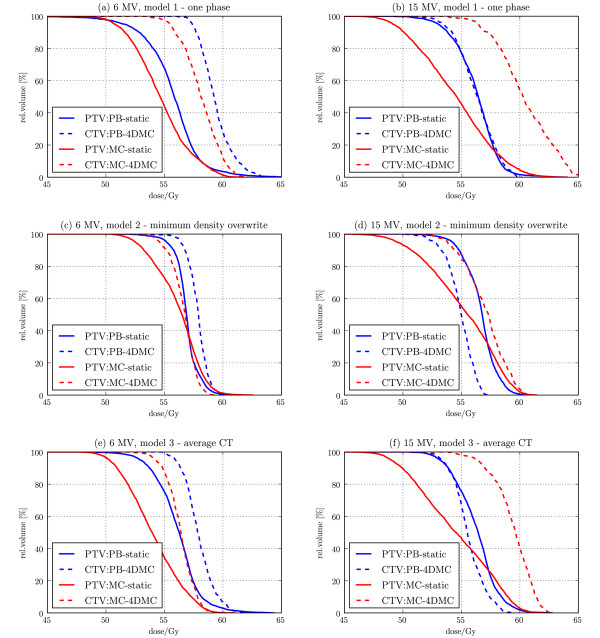
**Target dose distribution DVHs**. Target (PTV) DVHs of the PB and MC static plans (solid) and their 4DMC calculation (CTV accumulated dose DVHs) (dashed). Calculated for 6 MV (left column) and 15 MV (right column) for the one phase static CT (a, b); the minimum density overwrite one phase static CT (c, d) and the average CT (e, f) patient models.

**Figure 7:**
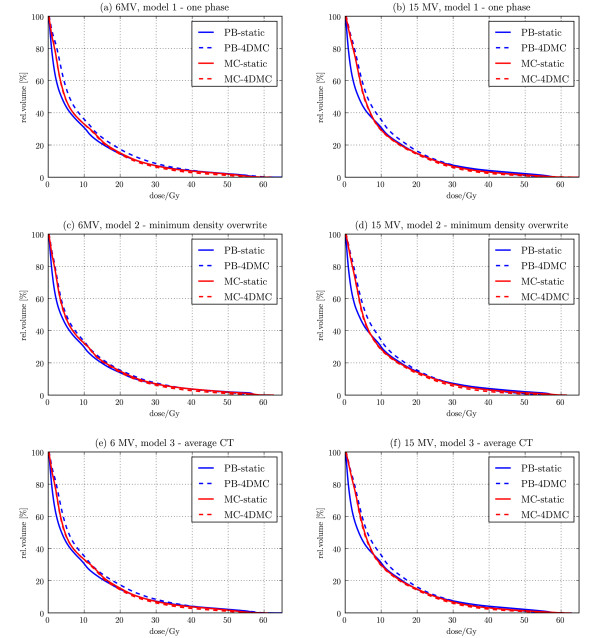
**Ipsilateral lung dose distribution DVHs**. Ipsilateral lung DVHs of the PB and MC static plans (solid) and their 4DMC calculation (accumulated dose DVHs) (dashed). Calculated for 6 MV (left column) and 15 MV (right column) for the one phase static CT (a, b); the minimum density overwrite one phase static CT (c, d) and the average CT (e, f) patient models.

The 4DMC recalculation showed that the MC calculated dose on the static patient models actually underestimated the target-EUD for all patient models and for both 6 and 15 MV, while OAR isoeffects/EUDs were comparable (Table 1). The largest difference between the target-EUD calculated with the static patient model compared to 4D MC was found for the *one phase static CT *patient model and the smallest difference was found for the *minimum density overwrite one phase static CT *model, independent of beam energy (Figure 6). The 4DMC recalculation of the fsPB optimized plans confirmed that most of the OAR constraints were violated and that the fsPB dose calculation algorithm underestimated the target-EUD in case of 6 MV and overestimated the target-EUD in case of 15 MV for all patient models for this specific patient. Figure 7 shows DVHs of the ipsilateral lung. For all patient models, PB-static plans consequently underestimated dose relatively to the 4DMC accumulated dose while MC-static plans agree very well with the 4DMC accumulated dose. Small discrepancies can be noticed for MC treatment plans in lung volume close to the tumor where the MC-static plans slightly overestimated the lung dose which is related to smearing out high dose isolines to larger volume around the target for the static patient models. A performance test of the PB and MC planning showed that the PB planning (PB beamlet allocation, PB beamlet optimization, PB dose segment weight and shape optimization and final PB dose calculation) takes less than 30 min while the MC planning (PB beamlet allocation, PB beamlet optimization, MC dose segment weight and shape optimization and final MC dose calculation) takes from 1 h to 1.5 h depending on the difficulty of converging to the plan constraints and the number of segments in the final plan.

## Discussion

In this study we have shown how dose calculation of small fields in the presence of tissue heterogeneities and static modelling of a moving target influence fsPB and MC dose calculation and optimization for lung IMSRT. The results of the lung IMSRT planning are presented for one extreme case (small tumor, large movements, density inhomogeneities) in order to emphasize possible dose calculation and patient model uncertainties and less extreme cases will produce smaller problems.

While the MC dose calculation performed well for a static lung phantom, the fsPB algorithm always underestimated the target dose compared to film measurements. This confirms what has been found by others for various superposition/convolution and collapsed cone algorithms [10,12,28,8,3,5,29]. The MC can safely be regarded as the superior algorithm for dose calculation on a static geometry.

When looking at real patient data of lung IMSRT, the picture gets more complicated and it is difficult to interpret the results from fsPB planning. Vanderstraeten et al. [29] investigated dose calculation with various commercially available conventional dose engines, and showed overestimation or underestimation of the target dose when comparing to the MC calculated dose, depending on the algorithm. Our results showed that our fsPB algorithm both underestimated and overestimated the target-EUD depending on the energy (6 or 15 MV). This is related to uncertainties in penumbra widening factors (especially parameter *f*_*u*1_(*ρ*)) of the fit functions implemented in the fsPB for low densities (Figure 4, Jeleń et al [22]). The dose to the ipsilateral lung calculated by the fsPB was underestimated for all static plans while MC static plans agreed very well with the 4DMC recalculations. Thus, for this patient example lung IMSRT treatment planning with the fsPB results in higher complication probability then MC-based planning.

In addition to an unpredictable target-EUD, optimization with fsPB led to violation of most OAR constraints for both low and high energy photon beams when compared to MC recalculated dose on both the static and 4D patient models. This means that optimization with fsPB and recalculation with MC is impractical, because the violated OAR constraints would have to be readjusted and the whole optimization procedure rerun. Bearing this in mind, optimization with fsPB and recalculation with MC may not necessarily be more efficient than MC based optimization which needed less than 1.5 hours in our case. Although MC is superior to fsPB on a static geometry, the real accumulated dose to the patient is influenced by the breathing motion during delivery. In our study, the 4D MC recalculations of the MC optimized treatment plans showed that the target doses calculated on the static patient models were always lower than the 4D accumulated target dose.

The differences between static and 4D recalculated MC dose depended on the patient model. The largest difference, as expected, was found for the *one phase static CT *model, where a large part of the target was occupied by low density lung tissue to where the optimizer tried to deliver a higher fluence in order to achieve a homogeneous target dose. The *minimum density overwrite one phase static CT *model worked well for our patient, especially for the 6 MV plan. The advantage of this patient model is that the optimizer is not miss-leaded by low densities in the target. In contrast, the *average CT *model still contains a significant fraction of low density lung tissue (like the *one phase static CT *model) to where the optimizer also tries to boost the dose but which is rarely visited by the tumor.

For the fsPB optimized plans, 4D MC recalculation displayed a complex picture where dose calculation uncertainties and geometrical uncertainties either added up or cancelled out. Because only geometrical uncertainties were present with the MC optimized plans, these were more predictable. Although always underestimating the target dose compared to 4D MC, the MC optimized plans showed only small deviations in the OAR isoeffects/EUDs, especially for relatively static organs. The 4DMC dose were higher because the dose "follows" the higher density of the tumor during breathing. We therefore consider MC based optimization on static patient models to be a safe method for lung IMSRT planning for the minimum density or average CT models tested in this study.

4D MC optimization was not considered in the present paper. It eliminates the uncertainties in the geometrical model and provides the optimal dose for free breathing IMSRT treatment with a negligible increase of optimization and calculation time at the price of a greater dependency on the consistency between predicted and realized patient breathing motion [20].

## Conclusion

The MC dose engine was superior to fsPB in presence of lateral electron disequilibrium on static geometries. With an efficient MC system, MC based optimization of lung IMSRT is feasible, also given clinical time constraints. MC dose optimization on static patient models always underestimated the CTV dose compared to 4DMC recalculations, but OAR differences were very small. Therefore, MC optimization of lung IMSRT using a static patient model is recommended.

## Competing interests

The authors declare that they have no competing interests.

## Authors' contributions

MSi participated in the design of the study, did the literature review and drafted the manuscript. JM performed the experimental work. MSö conducted the 4DMC recalculation. MW participated in the clinical lung IMSRT planning. MA conceived of the study, and participated in its design and coordination. All authors read and approved the final manuscript.
